# Association between non-high-density lipoprotein cholesterol to high-density lipoprotein cholesterol ratio and obstructive sleep apnea: a cross-sectional study from NHANES

**DOI:** 10.1186/s12944-024-02195-w

**Published:** 2024-07-04

**Authors:** Xue Pan, Xinyue Zhang, Xinyi Wu, Yue Zhao, Yilong Li, Zitong Chen, Yue Hu, Xuezhao Cao

**Affiliations:** Department of Anesthesiology, the First Hospital of China Medical University, Shenyang, China

**Keywords:** NHHR, NHANES, Obesity, Obstructive sleep apnea

## Abstract

**Background:**

Obstructive Sleep Apnea (OSA) is a widespread sleep disturbance linked to metabolic and cardiovascular conditions. The Non-High-Density Lipoprotein Cholesterol to High-Density Lipoprotein Cholesterol Ratios (NHHR) has been proposed as being a potential biomarker to gauge cardiovascular risk. However, its relationship with OSA remains unclear.

**Methods:**

This survey investigated the link NHHR to OSA in American citizens aged 20 and older using information collected via the National Health and Nutrition Examination Survey (NHANES) during the years 2017 to 2020. Logistic regression models with multivariable adjustments were employed to assess this relationship. Nonlinear associations were explored using smooth curve fitting, with a two-part linear regression model identifying a threshold effect. Subgroup analyses were conducted to evaluate population-specific differences.

**Results:**

The survey encompassed 6763 participants, with an average age of 50.75 ± 17.32. The average NHHR stood at 2.74, accompanied by a standard deviation of 1.34, while the average frequency of OSA was 49.93%. Upon adjusting for covariates, each unit increase in NHHR may be associated with a 9% rise in OSA incidence. (95% confidence intervals 1.04–1.14; *P* < 0.0001). Notably, a U-shaped curve depicted the NHHR-OSA relationship, with an inflection point at 4.12. Subgroup analyses revealed consistent associations, with educational attainment and diabetes status modifying the NHHR-OSA relationship.

**Conclusion:**

The study highlights NHHR as a potential tool for OSA prediction, presenting avenues for advanced risk evaluation, tailored interventions, personalized treatment approaches, and preventive healthcare.

**Supplementary Information:**

The online version contains supplementary material available at 10.1186/s12944-024-02195-w.

## Introduction

Obstructive Sleep Apnea (OSA) is a prevalent sleep disorder characterized by recurrent episodes of upper airway obstruction during sleep, leading to sporadic hypoxemia and recurrent awakenings. In the United States (US), it affects roughly 17% of women and 34% of men aged 30 and 70 [1]. Symptoms of OSA include excessive daily sleepiness, neurocognitive impairment [2], diminished quantity of life, as well as endocrine, metabolic [3], and cardiovascular-related changes. Left untreated, OSA can precipitate serious health complications, including high blood pressure, cardiovascular diseases [4], metabolic syndrome [5] and diabetes [6]. Crucially, OSA has a significant relationship with changes in metabolism and is recognized as an independent risk factor for cardiovascular ailments. Therefore, there is an imperative to identify novel and more precise biomarkers for predicting the probability of adverse cardiovascular events in individuals afflicted by OSA.

Numerous studies have investigated the intricate connection between lipid abnormalities and OSA. For instance, a prospective cohort study involving 846 older adults demonstrated a robust association between high-density lipoprotein cholesterol (HDL-C) and severe OSA, while low-density lipoprotein (LDL) did not exhibit an independent association [7]. Similarly, a retrospective analysis of 2361 people revealed that, in comparison to controls, those with OSA had increased triglycerides, greater Non-High-Density Lipoprotein Cholesterol (NHDL-C) and lower HDL-C. Moreover, a significant link was observed with the severity of OSA of HDL-C [8]. Furthermore, individuals diagnosed with OSA exhibit notably increased levels of oxidized low-density lipoprotein (oxLDL), a factor associated with preclinical atherosclerosis [9]. HDL-C ameliorates the inhibitory effect of oxLDL on vascular reactivity [10].

The NHHR, integrating features of both HDL-C and non-HDL-C, has been recognized as a comprehensive marker for evaluating atherosclerosis. Previous research has demonstrated its superior predictive and diagnostic efficacy in assessing the risk of atherosclerosis [11], diabetes type 2 [12], and metabolism syndrome [13] compared to traditional lipid indicators. Furthermore, recent research has emphasized the linkage and prognostic worth of NHHR with different illnesses, including depression [14], kidney stones [15], and suicidal ideation [16]. Exploring the relationship between NHHR and OSA could yield valuable insights into the interconnectedness of lipid metabolism and sleep quality, potentially informing preventive and therapeutic strategies for these conditions.

The complexity of OSA and its implications for cardiovascular risk underscores the importance of identifying reliable biomarkers. Given this context, delving into the correlation between NHHR and OSA could unveil a simple yet effective tool for predicting OSA risk. Therefore, this study endeavors to explore the relationship between NHHR and OSA risk in the adult population. By elucidating this intricate connection, this study contributes to the existing knowledge by proposing NHHR as a comprehensive biomarker for evaluating OSA risk, offering advantages over traditional lipid parameters, such as HDL-C and LDL-C. The gleaned observations could pave the way for personalized management strategies and interventions to mitigate the adverse health outcomes associated with OSA.

## Methodology

### Study design and population

**Fig. 1:**
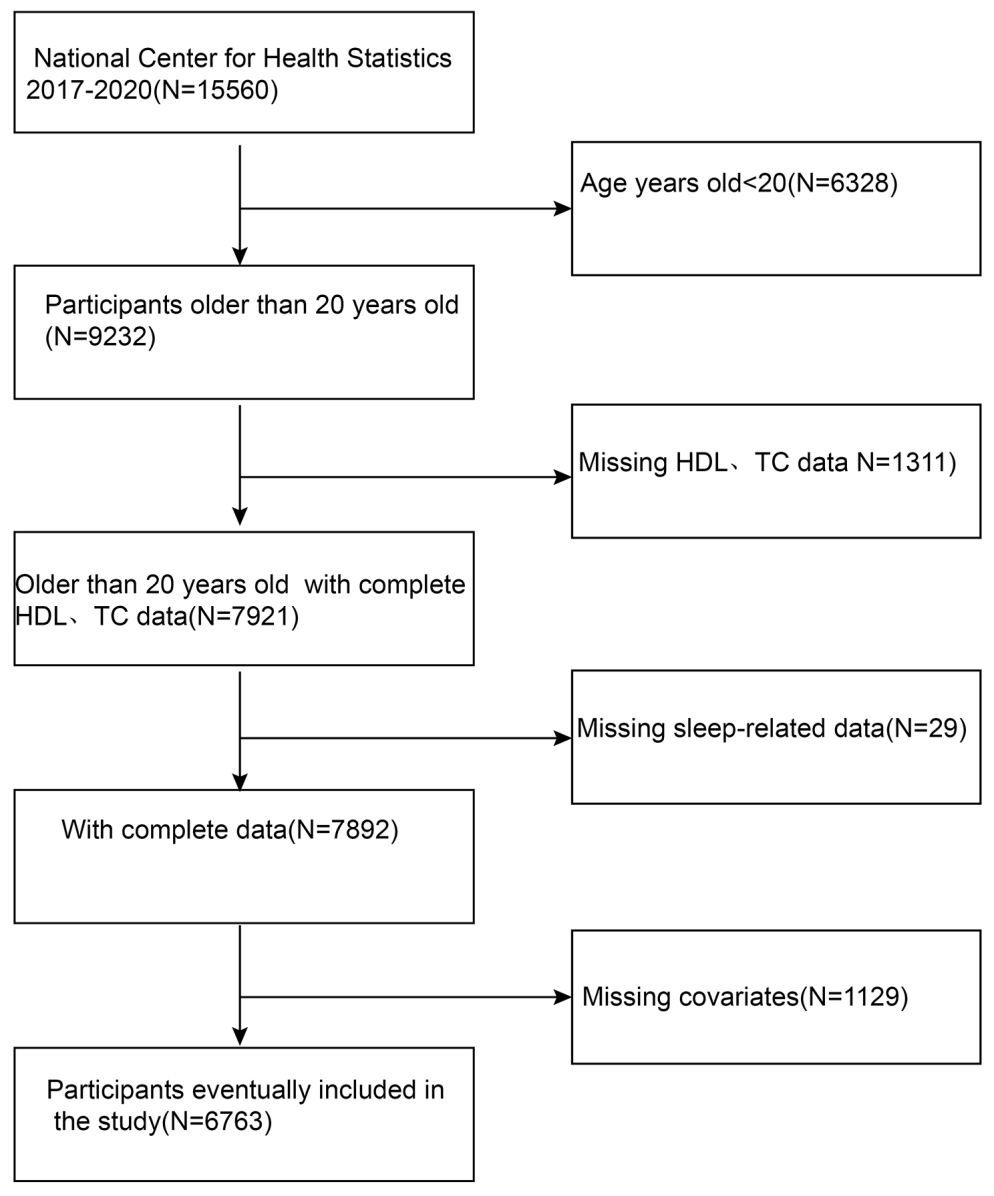
Flowchart of participant selection. HDL, High-Density Lipoprotein Cholesterol; TC, Total Cholesterol

This snapshot survey examines the link to NHHR and OSA, utilizing the data provided from the NHANES during the years 2017 to 2020. NHANES offers a comprehensive and accurate representation of the whole US population, providing in-depth information on health, nutrition, and demographic characteristics. The NHANES survey methodology involves a complex, multi-stage, probability-cluster sampling technique. Additional details regarding NHANES can be available on the webpage www.cdc.gov/nchs/Nhanes/. The entire NHANES participants provided informed signed agreement, and it obtained approval from the Research Ethics Committee of the National Centre for Health Statistics. The initial sample consisted of 15,560 individuals with valid NHANES data from 2017 to 2020. Exclusion criteria included individuals under 20 years old, those with missing NHHR or OSA data, and those with missing covariate data. The flowchart in Fig. 1 illustrates the specific selection process.

### Calculation of NHHR Index

The NHHR index was computed using the following calculation: (Total Cholesterol (TC) - HDL-C) split by HDL-C.

### Diagnosis of OSA

High-risk for OSA was defined based on participant responses to three binary questions in NHANES: (1) Suffering chronic extreme daytime sleepiness, even after getting around 7 h or more of sleep per night on weekdays or workdays, happening 16–30 times each month; (2) Reporting breathing pauses, snorting, or gasping for air on 3 or more nights per week; (3) Loud snoring on 3 or more nights per week [17].

### Covariates

A multivariable-adjusted model was employed to summarize variables potentially influencing NHHR index and OSA correlation. Covariates considered in this study included various demographic and health-related factors, as outlined in Table 1 [17]. Detailed information regarding the measuring techniques for these variables is available on the official Centers for Disease Control and Prevention website at www.cdc.gov/nchs/nhanes/.

**Table 1:**
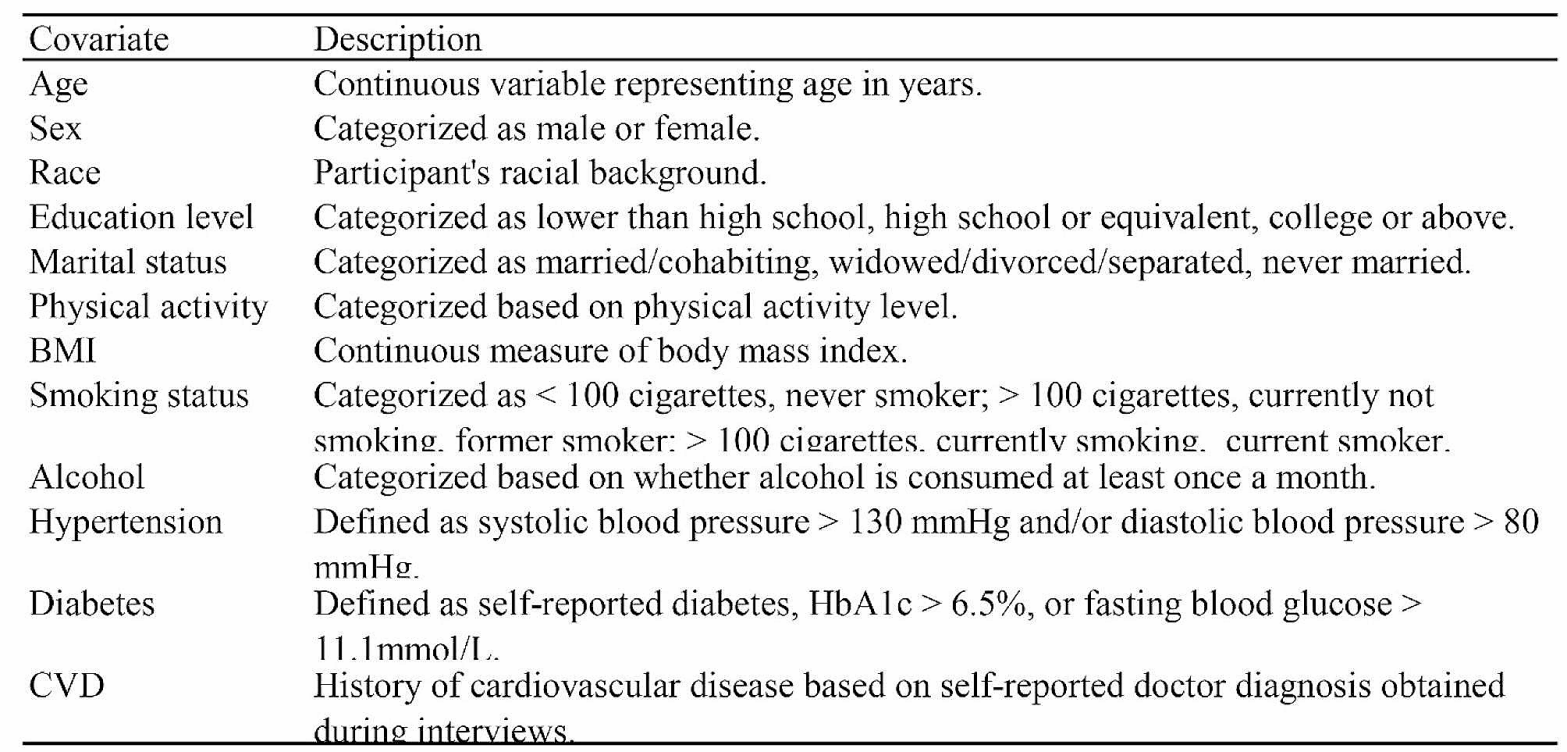
Covariates along with their descriptions or categorizations. Covariates included in this study. BMI, Body Mass Index; CVD, Cardiovascular Disease

### Statistical analysis

The analytical methods were implemented with R 4.0.5 software and Empowerstats 2.0, taking into consideration the intricate NHANES sampling design with sample visit weights. Descriptive analyses were conducted, reporting proportion adjusted for weights (%) of qualitative parameters and weighted averages with corresponding statistical dispersion pertaining to quantitative data. Qualitative parameters were assessed using chi-square tests, while quantitative data were investigated using analysis of variance (ANOVA).

Multivariable Logit models (Model I, II, and III) were developed to determine the odds ratio (OR) and 95% confidence intervals (CI) for the association between the NHHR index and OSA. Model I was unadjusted for covariates. Model II was adjusted for race, sex and age. Model III additionally included modifications for relationship situation, educational attainment, physical activity, BMI, smoking habits, drinking habits, high blood pressure, diabetes, and previous cardiovascular events.

Subgroup analyses and interaction tests were conducted to explore potential differences among the different populations. The study investigated the presence of nonlinear relationships between OSA and NHHR index by employing smooth curve fitting. Threshold relationships were investigated using a two-part linear regression. The predictive capacity of NHHR, HDL-C, and TC for the incidence of OSA was evaluated using Receiver Operating Characteristic (ROC) curves and Area Under the Curve (AUC). A *P*-value less than 0.05 was considered statistically significant for all results.

## Results

### Baseline characteristics of participants

The paper’s survey comprised 6,763 participants, with an average age of 50.75 ± 17.32 years. The sample consisted of 48.38% males and 51.62% females. Table 2 presents the weighted baseline traits. The mean NHHR index was 2.74 ± 1.34. NHHR quartile ranges were defined as follows: Quartile 1 included values less than or equal to 1.82, Quartile 2 encompassed values between 1.82 and 2.50, Quartile 3 ranged between 2.51 and 3.38, and Quartile 4 comprised values greater than 3.38. The prevalence of OSA was 49.93% among participants. Quartile 4 of NHHR, when compared to Quartile 1, exhibited likelihoods with older age, a greater percentage of males, lower levels of education, higher BMI, increased rates of smoking, elevated rates of high blood pressure and diabetes, lower HDL-C, and higher TC.

**Table 2:**
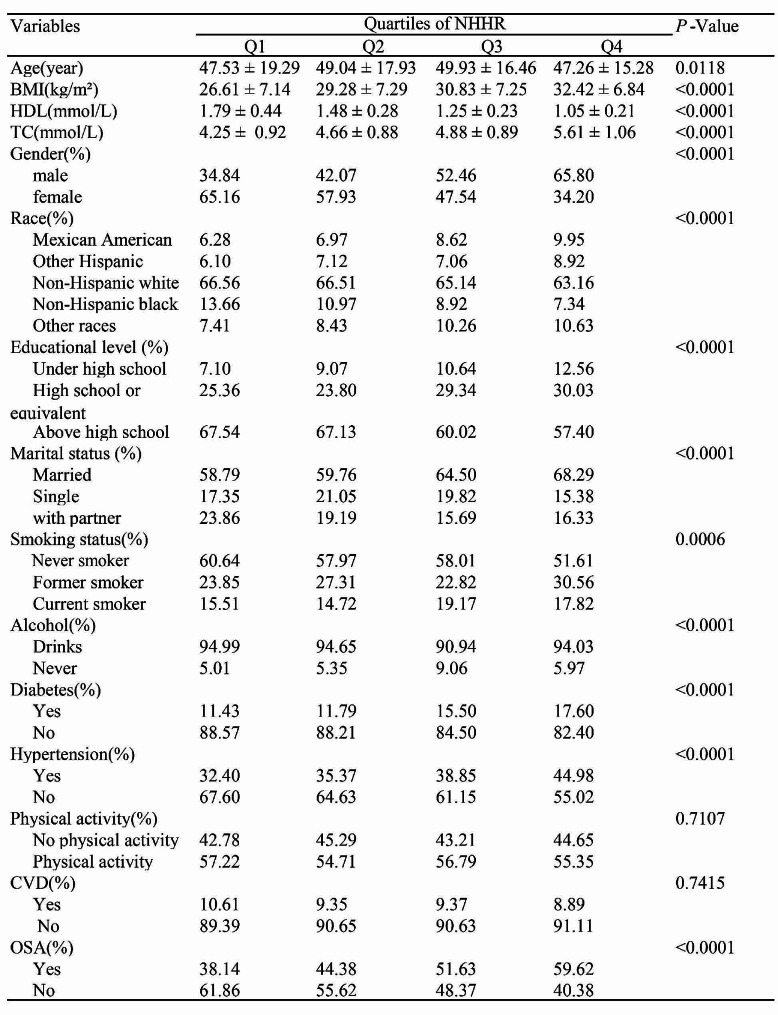
Baseline characteristics of participants in the NHANES 2017–2020. Categorized according to NHHR quartiles. NHHR, Non-high-density Lipoprotein Cholesterol to High-Density Lipoprotein Cholesterol Ratio; BMI, body Mass Index; HDL, High-Density Lipoprotein Cholesterol; TC, total cholesterol; CVD, Cardiovascular Disease; OSA, Obstructive Sleep Apnea

### Association between NHHR Index and OSA Risk

After adjusting for all relevant factors, each additional unit of NHHR showed a 9% positive correlation with the occurrence of OSA (OR = 1.09; 95% CI 1.04–1.14; *P* < 0.01). This indicates a strong positive association between NHHR and OSA. Furthermore, analysis of NHHR in quartiles revealed that Quartile 4 had a 38% higher likelihood of OSA compared to Quartile 1 (OR = 1.38; 95% CI 1.19–1.61; *P* < 0.01) (Table 3).

**Table 3:**
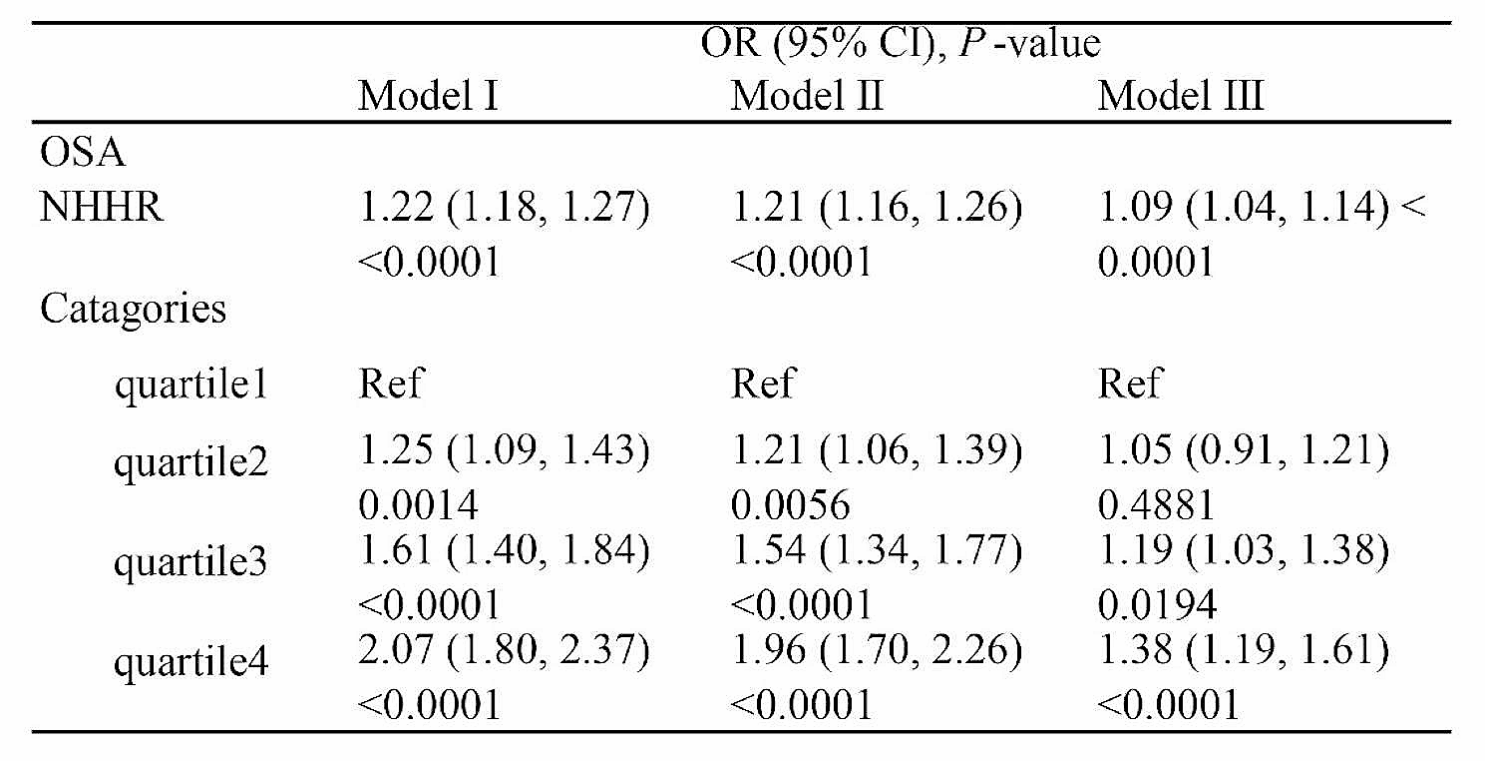
Multivariable logistic regression models for the association between NHHR and OSA

In the analysis, NHHR was examined as a continuous variable and a categorical variable in quartiles.

Model I, no covariates were adjusted. Model II, Age, Gender, and Race were adjusted. Model III, Age, Gender, Race, Marital status, Education level, Physical activity, BMI, Smoke status, Alcohol, Hypertension, Diabetes, and History of cardiovascular disease. OR, Odds Ratio; CI, Confidence Intervals; OSA, Obstructive Sleep Apnea; NHHR, Non-High-Density Lipoprotein Cholesterol to High-Density Lipoprotein Cholesterol Ratio; Ref, Reference.

### Analysis of curve fitting and threshold effects

**Fig. 2:**
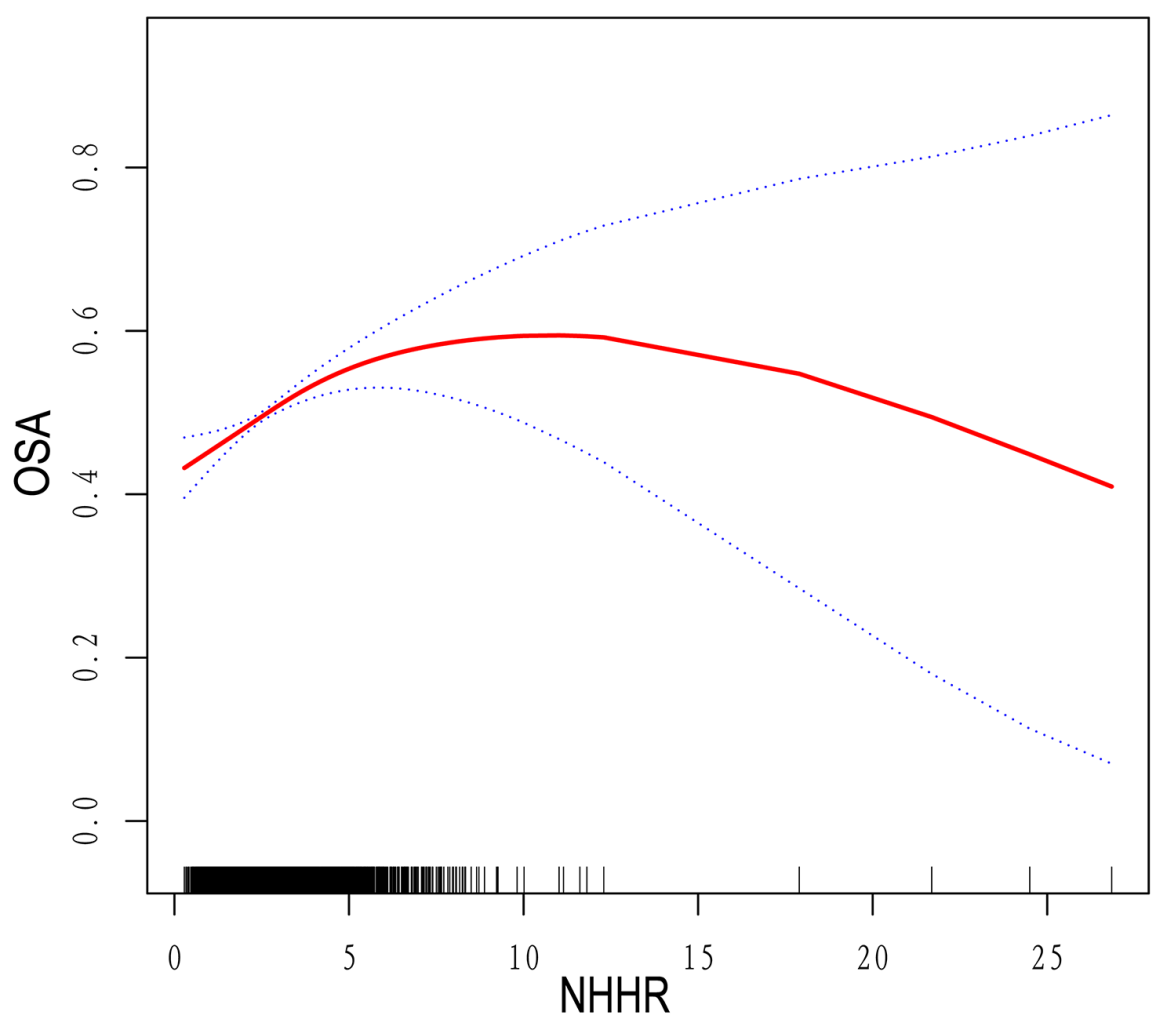
Smoothed curve fit between NHHR and OSA The blue bars show the fitted 95% confidence intervals (95% CI) and the fitted smoothed curves are shown in red. OSA, Obstructive Sleep Apnea; NHHR, Non-High-Density Lipoprotein Cholesterol to High-Density Lipoprotein Cholesterol Ratio

Model III revealed an inverted U-shaped relationship between NHHR index and OSA prevalence (Fig. 2**)**. Further analysis of threshold effects revealed a curve inflection point at 4.12. When NHHR falls below this threshold, each additional unit increase is associated with a 16% higher risk of OSA (OR = 1.16; 95% CI 1.09–1.23; *P* < 0.0001). However, values above 4.12 did not yield significant results in their relationship (OR = 0.98; 95% CI 0.91–1.06; *P* = 0.5986), as indicated by a *P*-value of 0.004 from the likelihood ratio test (Table 4).

**Table 4:**
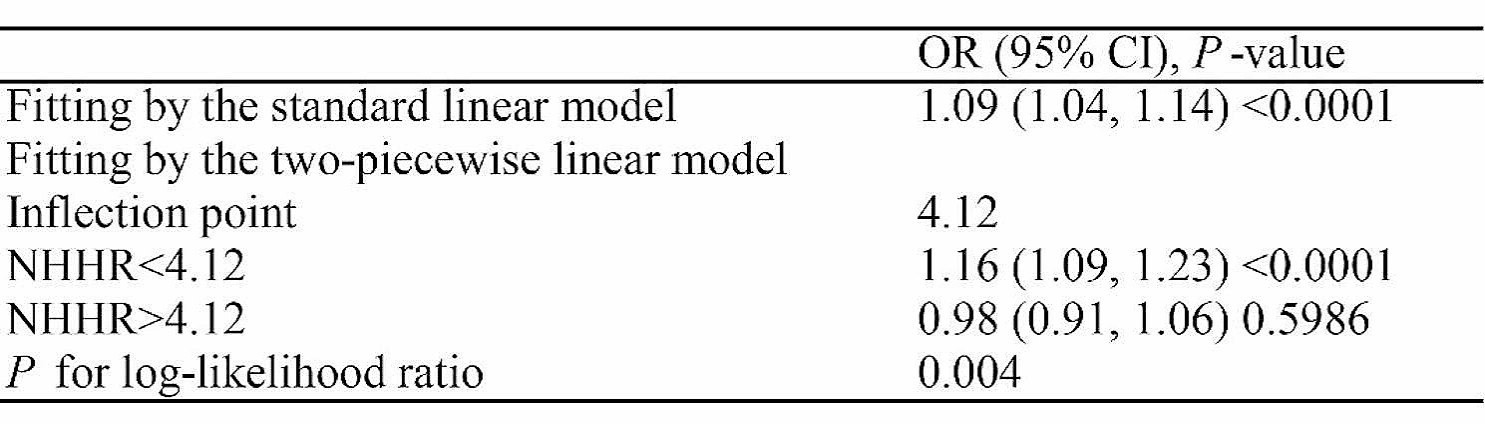
The threshold effect analysis of the NHHR on OSA risk. OR, odds ratio; CI, confidence intervals; NHHR, Non-high-density Lipoprotein Cholesterol to High-Density Lipoprotein Cholesterol Ratio; OSA, Obstructive Sleep Apnea

### Subgroup Analysis

Multi-subgroup analyses and interaction tests, based on various covariates, were conducted to assess the strength of the NHHR-OSA relationship and identify potential population differences (Table 5). In most subgroups, a consistent association between NHHR and OSA was observed. However, educational attainment was found to modify this association (*P* for interaction = 0.0016). Furthermore, differences were noted among diabetic populations (*P* for interaction = 0.0129), with a stronger NHHR-OSA association observed in non-diabetic individuals (OR = 1.11; 95% CI 1.05–1.16; *P* < 0.0001), compared to diabetic patients (OR = 0.98; 95% CI 0.91–1.06; *P* = 0.6789).

**Table 5:**
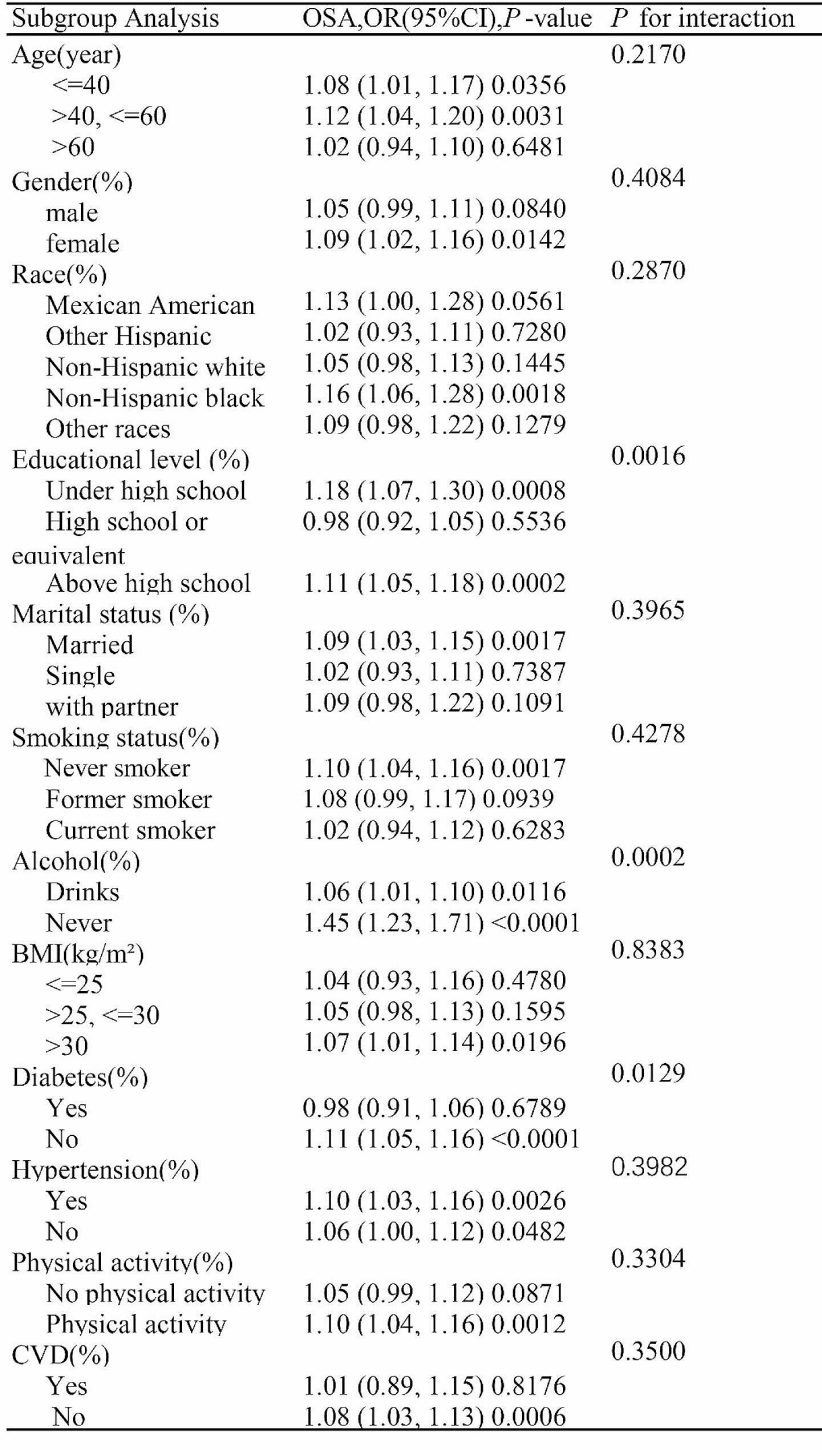
Stratified analysis of the correlation between NHHR and OSA. Stratification of covariates adjusted according to model III. OR, odds ratio; CI, confidence intervals; OSA, Obstructive Sleep Apnea; BMI, body Mass Index; CVD, Cardiovascular Disease; NHHR, Non-high-density Lipoprotein Cholesterol to high-density lipoprotein cholesterol ratio

### ROC curve analysis

**Fig. 3:**
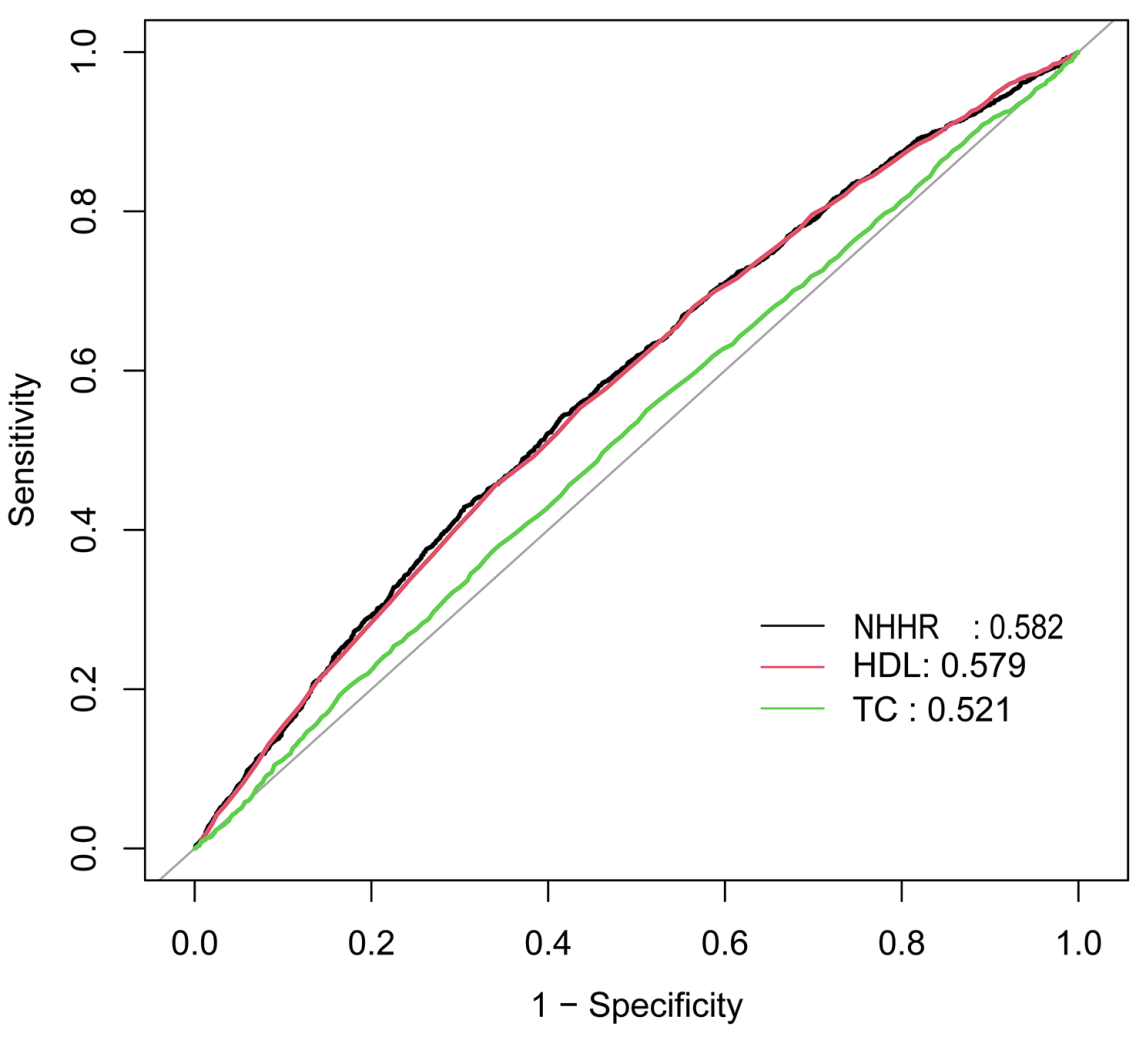
ROC curve between OSA and NHHR. ROC, Receiver Operating Characteristic; OSA, Obstructive Sleep Apnea; NHHR, Non-High-Density Lipoprotein Cholesterol to High-Density Lipoprotein Cholesterol Ratio; HDL, High-Density Lipoprotein Cholesterol; TC, Total Cholesterol

ROC curve analysis indicated that NHHR exhibited slightly higher specificity compared to HDL-C and TC, with a specificity of 0.5839 and sensitivity of 0.5434 (Fig. 3).

### Discussion

This cross-sectional research encompassed a representative sample of 6,763 American citizens aged 20 and older from the NHANES dataset. The analysis revealed an essential connection between NHHR and the likelihood of OSA. This robust association persisted even following adjusting for various covariates, suggesting that NHHR could serve as a reliable tool for assessing the risk of OSA. The subgroup analysis revealed that individuals with educational attainment levels below high school or above college should pay particular attention to managing NNHR. Notably, these findings revealed a nonlinear pattern, with a critical threshold identified at an NHHR of 4.12, beyond which the association with OSA risk diminished. These results underscore the clinical relevance of maintaining an optimal NHHR level to potentially mitigate the risk of OSA.

The findings contribute to the expanding body of evidence linking lipid abnormalities, specifically NHHR, to the risk of developing OSA. Previous investigations have primarily focused on the roles of HDL-C and TC concerning OSA. The Sleep Heart Health Study research group conducted a prospective cohort study that identified a robust association between HDL-C levels and the severity of sleep-disordered breathing [18]. Similarly, a recent case-control study involving 1,310 children observed lower HDL-C levels and higher TC in OSA patients [19]. Animal models have also suggested that intermittent hypoxia characteristic of OSA may increase HDL-C expression, potentially through the upregulation of the HDL-C receptor SRB1 [20]. These studies indirectly support the findings, indicating a complex interplay between lipid metabolism and OSA pathogenesis.

However, conflicting results have emerged from various studies, highlighting the need for further investigation. For instance, a cross-sectional study involving 753 Australian men revealed no significant correlation between OSA and HDL-C levels [21]. Furthermore, a Mendelian randomization (MR) investigation revealed no clear correlation between HDL-C and OSA [22]. These discrepancies may arise from variations in study populations, ethnicities, and OSA assessment criteria. Thus, this study aimed to introduce NHHR as a novel atherosclerosis indicator, potentially enhancing the predictive ability of HDL-C and non-HDL-C for OSA risk.

The connection regarding NHHR and OSA incidence can be explained by various mechanisms incurred by lipid metabolism. Dysfunctional HDL-C, particularly its subfractions HDL1-3, is implicated in atherosclerosis [23]. HDL-C’s anti-inflammatory, antioxidant, and anti-atherosclerotic properties are crucial in this context [24]. HDL-C enhances endothelial function through various mechanisms, including raising intracellular Ca^2+^ levels, activating Akt to trigger the release of nitric oxide, and increasing the expression of endothelial nitric oxide synthase (eNOS) via lysophospholipids, such as sphingosylphosphorylcholine (SPC), sphingosine-1phosphate (S1P), and lysosulfatide (LSF) [25, 26]. Additionally, HDL enhances the expression of eNOS by interacting with SRB1 receptors present on endothelial cells [27]. Dysfunctional HDL-C may also contribute to increased levels of oxLDL, promoting inflammation and atherosclerosis [28].

The clinical relevance of this study lies in its identification of NHHR as a potential biomarker for predicting OSA risk in adults. This finding has direct implications for patient care across several domains: (1) Enhanced Risk Assessment: NHHR demonstrates advantages over traditional lipid parameters in predicting OSA risk. Integrating NHHR measurements into routine assessments enables clinicians to more accurately identify individuals prone to OSA development. (2) Targeted Interventions: Recognizing NHHR as a predictive biomarker for OSA enables healthcare providers to implement focused interventions aimed at managing lipid irregularities to mitigate OSA risk. These interventions may involve tailored lifestyle modifications, pharmacotherapy, or other targeted approaches based on individual patient profiles. (3) Personalized Management Strategies: Understanding NHHR’s role in OSA pathophysiology opens avenues for personalized management strategies. Clinicians can utilize NHHR measurements to customize treatment plans and interventions for patients with OSA, potentially leading to improved outcomes and enhanced patient care. (4) Preventive Medicine: NHHR may help predict the occurrence of OSA, allowing for preventive measures to be initiated before significant symptoms or complications emerge. Early identification and intervention hold the potential to prevent or delay OSA progression and its associated cardiovascular risks, promoting overall health and well-being.

### Study strengths and limitations

The conclusion drawn from this study is robust, as it involved a large and geographically representative group of US adults from NHANES, with comprehensive adjustments made for covariates. However, certain limitations should be acknowledged. The nature of this snapshot survey investigation precludes the establishment of a direct causal link between NHHR and OSA, and the potential for reverse causality cannot be entirely ruled out. Additionally, the cholesterol data utilized were obtained from fasting samples, which may differ from non-fasting levels. Objective indicators for OSA evaluation were limited, and the study did not extend to investigating NHHR and OSA in children.

### Conclusion

In conclusion, this study highlights the importance of NHHR as a predictive biomarker for OSA, offering opportunities for enhanced risk assessment, targeted interventions, personalized management strategies, and preventive medicine. Integration of NHHR assessments into clinical routines enables healthcare professionals to enhance patient care by identifying those susceptible to OSA and initiating proactive measures to.

alleviate this risk, consequently enhancing overall health outcomes.

### Electronic supplementary material

Below is the link to the electronic supplementary material.

Supplementary Material 1

## Data Availability

Data supporting the findings of this study are available within the manuscript .

## Abbreviations

NHDL-C: Non-high-density lipoprotein cholesterol
HDL-C: High-density lipoprotein cholesterol
LDL: low-density lipoprotein
oxLDL: oxidized low-density lipoprotein
TC: Total Cholesterol
NHHR: NHDL-C and HDL-C ratio
OSA: Obstructive Sleep Apnea
NHANES: National Health and Nutrition Examination Survey
BMI: Body mass index
CVD: Cardiovascular Disease
